# Association of genetic variation in the *NR1H4* gene, encoding the nuclear bile acid receptor FXR, with inflammatory bowel disease

**DOI:** 10.1186/1756-0500-5-461

**Published:** 2012-08-28

**Authors:** Ragam Attinkara, Jessica Mwinyi, Kaspar Truninger, Jaroslaw Regula, Pawel Gaj, Gerhard Rogler, Gerd A Kullak-Ublick, Jyrki J Eloranta

**Affiliations:** 1 Department of Clinical Pharmacology and Toxicology, University Hospital, Zurich, Switzerland; 2 Department of Biomedicine, Institute of Biochemistry and Genetics, University of Basel, Switzerland and Division of Gastroenterology, Regional Hospital, Langenthal, Switzerland; 3 Department of Gastroenterology and Hepatology, Medical Center for Postgraduate Education and the Maria Skłodowska Curie Memorial Cancer Center, Institute of Oncology, Warsaw, Poland; 4 Division of Gastroenterology and Hepatology, University Hospital, Zurich, Switzerland

**Keywords:** Bile acid homeostasis, Crohn’s disease, Farnesoid X receptor, Inflammatory bowel disease, Single nucleotide polymorphisms, Ulcerative colitis

## Abstract

**Background:**

Pathogenesis of inflammatory bowel diseases (IBD), ulcerative colitis (UC) and Crohn’s disease (CD), involves interaction between environmental factors and inappropriate immune responses in the intestine of genetically predisposed individuals. Bile acids and their nuclear receptor, FXR, regulate inflammatory responses and barrier function in the intestinal tract.

**Methods:**

We studied the association of five variants (*rs3863377*, *rs7138843*, *rs56163822*, *rs35724*, *rs10860603*) of the *NR1H4* gene encoding FXR with IBD. 1138 individuals (591 non-IBD, 203 UC, 344 CD) were genotyped for five *NR1H4* genetic variants with TaqMan SNP Genotyping Assays.

**Results:**

We observed that the *NR1H4* SNP *rs3863377* is significantly less frequent in IBD cases than in non-IBD controls (allele frequencies: P = 0.004; wild-type vs. SNP carrier genotype frequencies: P = 0.008), whereas the variant *rs56163822* is less prevalent in non-IBD controls (allele frequencies: P = 0.027; wild-type vs. SNP carrier genotype frequencies: P = 0.035). The global haplotype distribution between IBD and control patients was significantly different (P = 0.003). This also held true for the comparison between non-IBD and UC groups (P = 0.004), but not for the comparison between non-IBD and CD groups (P = 0.079).

**Conclusions:**

We show that genetic variation in FXR is associated with IBD, further emphasizing the link between bile acid signaling and intestinal inflammation.

## Background

Inflammatory bowel disease (IBD) is a chronic condition characterized by recurrent inflammation of intestinal mucosa, and results from aberrant regulation of the mucosal immune system in genetically susceptible individuals. The etiology of IBD involves a complex interaction of genetic, environmental, and immunomodulatory factors. Two major forms of chronic mucosal inflammation have been defined. In Crohn’s disease (CD), the whole gastrointestinal tract may be affected, although the most frequent site of inflammation is the terminal ileum, whereas in ulcerative colitis (UC) the mucosal inflammation typically affects the colon [1]. For CD pathogenesis, a strong genetic component has been suggested by the concordance of 63.6% in monozygotic twins, but only of 3.6% in dizygotic twins. The concordance of monozygotic twins is lower (6%) in UC, indicating that genetic susceptibility may play a somewhat smaller role in this disease [2].

Nuclear receptors are a large family of transcription factors that are involved in the regulation of numerous processes, including reproduction, development, and a wide range of metabolic pathways [3]. The ligand-dependent activation function at the carboxy-terminus of most nuclear receptors allows them to sense metabolic changes within cells, and orchestrate rapid transcriptional changes in response [4-6].

The farnesoid X receptor (FXR; gene symbol *NR1H4*) is a nuclear receptor that functions as the main sensor of intracellular bile acid levels [7-9]. The human *NR1H4* gene is located on chromosome 12 and is composed of 11 exons and 10 introns [10]. The translation initiation codon of the *NR1H4* gene lies at the 3^′^ end of exon 3, whereas exons 1 and 2, together with the 5^′^ region of exon 3, contain the 5^′^ untranslated region (5’-UTR). Multiple FXR isoforms can be generated via alternative promoter usage and alternative splicing, and these isoforms may have differential transactivation abilities on specific target promoters [11]. FXR typically acts by binding to FXR response elements within the target promoters as heterodimers with another member of the nuclear receptor family, retinoid X receptor-α (RXRα) [12]. In response to elevated levels of intracellular bile acids, activated FXR is well known to induce protective gene expression circuits against bile acid toxicity in the liver and intestine [13]. Expression of bile acid efflux systems in ileocytes (organic solute transporter α/β; OSTα/β) [14,15] and hepatocytes (bile salt export pump; BSEP) [16-18] is upregulated by bile acid-activated FXR, while the expression of the respective bile acid uptake systems apical sodium-dependent bile acid transporter (ASBT) [19] and Na^+^-taurocholate cotransporting polypeptide (NTCP) [20,21] is suppressed by it. FXR also represses transcription of three genes coding for bile acid synthesizing enzymes, namely cholesterol-7α-hydroxylase (CYP7A1), sterol-12α-hydroxylase (CYB8B1), and sterol-27-hydroxylase (CYP27A1) [22,23]. Thus, elevated levels of bile acids can suppress their own *de novo* production through a negative feedback loop involving FXR. In addition, FXR regulates several genes that can protect against intestinal inflammation and bacterial overgrowth [24-26]. Fxr-deficient mice have increased ileal concentrations of gut bacteria and exhibit defects in the integrity of the intestinal epithelial barrier. In agreement with this, the products of a number of genes that are regulated by Fxr in the ileum, including angiogenin (*Ang1*), inducible nitric oxide synthase (*iNos*), and interleukin-18 (*Il-18*), are known to have antimicrobial actions [26]. Furthermore, it has been reported that reduced expression of Fxr/FXR is associated with colon inflammation in rodent models of colitis and in CD patients [25]. Recently, FXR activation was shown to decrease NF-κB-mediated immune responses and intestinal permeability in mouse models of colitis [27]. It was subsequently shown that intestinal inflammation reduces FXR activation as well as the expression of FXR target genes such as intestinal bile acid-binding protein (*IBABP*) and fibroblast growth factor 15/19 (*FGF15/19*) [28]. In agreement with this, it has been proposed that FXR may contribute to the resistance of both human and mouse gastric epithelial cells against inflammation-induced injury [29]. The fact that FXR thus appears to play a role in the protection of the integrity of the intestinal epithelial barrier and its inverse correlation with the level of intestinal inflammation suggest a potential connection between FXR and the molecular pathogenesis of IBD. FXR variants have been previously studied in association with several liver diseases, such as gallstone disease [30], cholangiocarcinoma [31], intrahepatic cholestasis of pregnancy [32], and idiopathic infantile cholestasis [33]. Here, we have investigated five *NR1H4* single nucleotide polymorphisms - two common SNPs and three rare variants - which have been previously studied in the context of human disease, in a well-sized IBD vs. non-IBD cohort, and report that two of these genetic variants are associated with IBD.

## Results

### Study population

The study population was European and consisted of more women (806) than men (332). Detailed demographic data is given in Table1.

**Table 1:** Demographic data of the population included in the analysis

**Characteristics**	**non-IBD**	**IBD**	**CD**	**UC**
**Population**	591 (51.9%)	547 (48.1%)	344 (30.2%)	203 (17.8%)
**Mean age** (± SD)	55.7 (± 12.6)	42.6 (± 15.1)	40.9 (± 14.9)	45.4 (± 14.9)
**Median age**	59	41	39	44
**Minimum age**	20	16	16	18
**Maximum age**	81	82	79	82

### *NR1H4* sequence variability

All five *NR1H4* variants selected for the study are single nucleotide substitutions, previously identified within the *NR1H4* gene (http://www.ncbi.nlm.nih.gov/snp/). Three of these SNPs can be considered as rare variants: *rs3863377*, *rs56163822*, *rs7138843*, with reported minor allele frequencies (MAF) of 4%, 2.2%, and 0.9%, respectively. The other two variants, *rs10860603* and *rs35724*, are common SNPs, with minor allele frequencies of 20.5% and 40.8% (http://www.ncbi.nlm.nih.gov/snp/) in European individuals. The genotype frequencies in all groups were in Hardy-Weinberg equilibrium (represented by the *χ*^2^ values in Table2). The obtained allele and genotype frequencies are given in Tables 3 and 2, respectively.

**Table 2:** **Genotype frequencies of the *NR1H4 *variants in the study population and the genotype association analysis**

**SNP *rs3863377*(G→ A)**								
	WT [GG]	Het [GA]	Hom [AA] (%)	P	P’	OR	CI	*χ*^2^
non-IBD	506 (94.4)	28 (5.2)	2 (0.4)					0.14
IBD	438 (97.8)	10 (2.2)	0	0.009** †	0.022*	0.39	0.19-0.80	0.01
CD	271 (97.8)	6 (2.2)	0	0.030* †	0.031*	0.37	0.15-0.191	0.01
UC	167 (97.7)	4 (2.3)	0	0.100 †	0.066	0.40	0.14-1.16	0.01
**SNP *rs56163822*(−1G → T)**								
	WT [GG]	Het [GT]	Horn [TT] (%)	P	P’	OR	CI	*χ*^2^
non-IBD	560 (96.6)	20 (3.4)	0					0.04
IBD	505 (93.9)	33 (6.1)	1 (0.09)	0.035*	0.451	1.83	1.04-3.23	0.00
CD	319 (94.4)	19 (5.6)	1 (0.09)	0.115	0.846	1.67	0.88-3. 17	0.00
UC	186 (93.0)	14 (7.0)	0	0.034*	0.154	2.11	1.04-4.26	0.13
**SNP *rs7138843*(A → T)**								
	WT [AA]	Het [AT]	Horn [TT] (%)	P	P’	OR	CI	*χ*^2^
non-IBD	495 (96.7)	17 (3.3)	0					0.03
IBD	417 (95.2)	21 (4.8)	0	0.248	0.772	147	0.76-2.82	0.06
CD	261 (96.0)	11 (4.0)	0	0.369 †	0.612	1.23	0.57-2.66	0.04
UC	156 (94.0)	10 (6.0)	0	0.097 †	0.981	1.87	0.84-4.16	0.10
**SNP *rs10860603*(G → A)**								
	WT [GG]	Het [GA]	Horn [AA] (%)	P	P’	OR	CI	*χ*^2^
non-IBD	424 (72.8)	143 (24.6)	15 (2.6)					049
IBD	407 (75.8)	121 (22.5)	9 (1.7)	0.261	0.384	0.86	0.66-1.12	0.00
CD	254 *(75.4)*	76 (22.5)	7 (2.1)	0.403	0.611	0.88	0.65-1.19	0.22
tiC	153 (76.5)	45 (22.5)	2 (1.0)	0.312	0.521	0.82	0.57-1.19	0.43
**SNP *rs35724*(G→ C)**								
	WT [GG]	Het [GC]	Horn [CC] (%)	P	P’	OR	CI	*χ*^2^
non-IBD	217 (37.3)	264 *(454)*	101 (17.3)					1.78
IBD	177 (32.9)	251 (46.7	110 (20.4)	0.125	0.209	1.213	0.95-1.55	1.47
CD	111 (32.8)	155 (45.9)	72 (21.3)	0.175	0.279	1.216	0.92-1.61	1.68
UC	66 (33.0)	96 (48.0)	38 (19.0)	0.277	0.233	1.207	0.86-1.69	0.09

**Table 3:** **Allele frequencies of the *NR1H4 *variants in the study population and the allele association analysis**

**SNP *rs3863377*(G → A)**					
**Case**	**MAF (%)**	**P**	**OR**	**CI**	**Reported MAFs**^**1**^
non-IBD	32 (2.9)				4.0%
IBD	10 (1.1)	0.004** †	0.37	0.18-0-75	
CD	6 (1.1)	0.015* †	0.36	0.15-0.86	
UC	4 (1.2)	0.075 †	0.38	0.14-1.10	
**SNP *rs56163822*, (G →T)**					
**Case**	**MAF (%)**	**P**	**OR**	**CI**	**Reported MAFs**^**1**^
non-IBD	20 (1.7)				2.2%
IBD	34 (3.2)	0.027*	1.86	1.06-3.25	
CD	20 (2.9)	0.081	1.74	0.93-3.25	
UC	14 (3.5)	0.036*	2.07	1.03-4.13	
**SNP *rs7138843*, (A → T)**					
**Case**	**MAF (%)**	**P**			**Reported MAFs**^**1**^
non-IBD	17 (1.7)				0.9%
IBD	21 (2.4)	ns			
CD	11 (2.0)	ns			
UC	10 (3.0)	ns	†		
**SNP *rs10860603*, (G → A)**					
**Case**	**MAF (%)**		**P**		**Reported MAFs**^**1**^
non-IBD	173 (14.9)				20.5%
IBD	139 (12.9)	ns			
CD	90 (13.4)	ns			
UC	49 (12.3)	ns			
**SNP *rs35724*, (G → C)**					
**Case**	**MAF (%)**	**P**			**Reported MAFs**^**1**^
non-IBD	466 (40.0)				40.8%
IBD	471 (43.8)	ns			
CD	299 (44.4)	ns			
UC	172 (43.0)	ns			

### Genetic variation in the *NR1H4* gene and IBD

The *NR1H4* SNP variants *rs3863377* and *rs56163822* were found to be significantly associated with IBD when considering an uncorrected significance level of p<0.05. Upon statistical analysis of the allele (Table3) and genotype (Table2) frequencies, we observed that the *NR1H4* variant *rs3863377* is significantly less frequent in IBD cases than in non-IBD controls (allele frequencies: P = 0.004; wild-type vs. SNP carrier genotype frequencies: P = 0.008) even when considering an Bonferroni-corrected significance level of P<0.01. Upon subgrouping the IBD patients, the significance of the inverse association of the *rs3863377* SNP remained for the CD patients when considering an uncorrected significance level of P<0.05 (allele frequencies: P = 0.015; wild-type vs. SNP carrier genotype frequencies: P = 0.024), but not for the UC group (allele frequencies: P = 0.075; wild-type vs. SNP carrier genotype frequencies: P = 0.083). Conversely, the variant *rs56163822* is less prevalent in non-IBD subjects than in IBD patients (allele frequencies: P = 0.027; wild-type vs. SNP carrier genotype frequencies: P = 0.035); an observation, which is, however, not significant, when considering a corrected significance level of P<0.01. Upon subgrouping the patient cohort according to IBD subtypes, the uncorrected association remained only significant for the UC group (allele frequencies: P = 0.036; wild-type vs. SNP carrier genotype frequencies: P = 0.034). Upon adjustment for age and gender, the uncorrected significance (P<0.05) of genotype frequency association with IBD remained for *rs3863377*, but was reduced to P>0.05 for *rs56163822*. There were no significant differences in the allele frequency distribution between the subject groups for *NR1H4* variants *rs7138843*, *rs10860603*, and *rs35724*.

### *NR1H4* haplotype analysis

**Figure 1:**
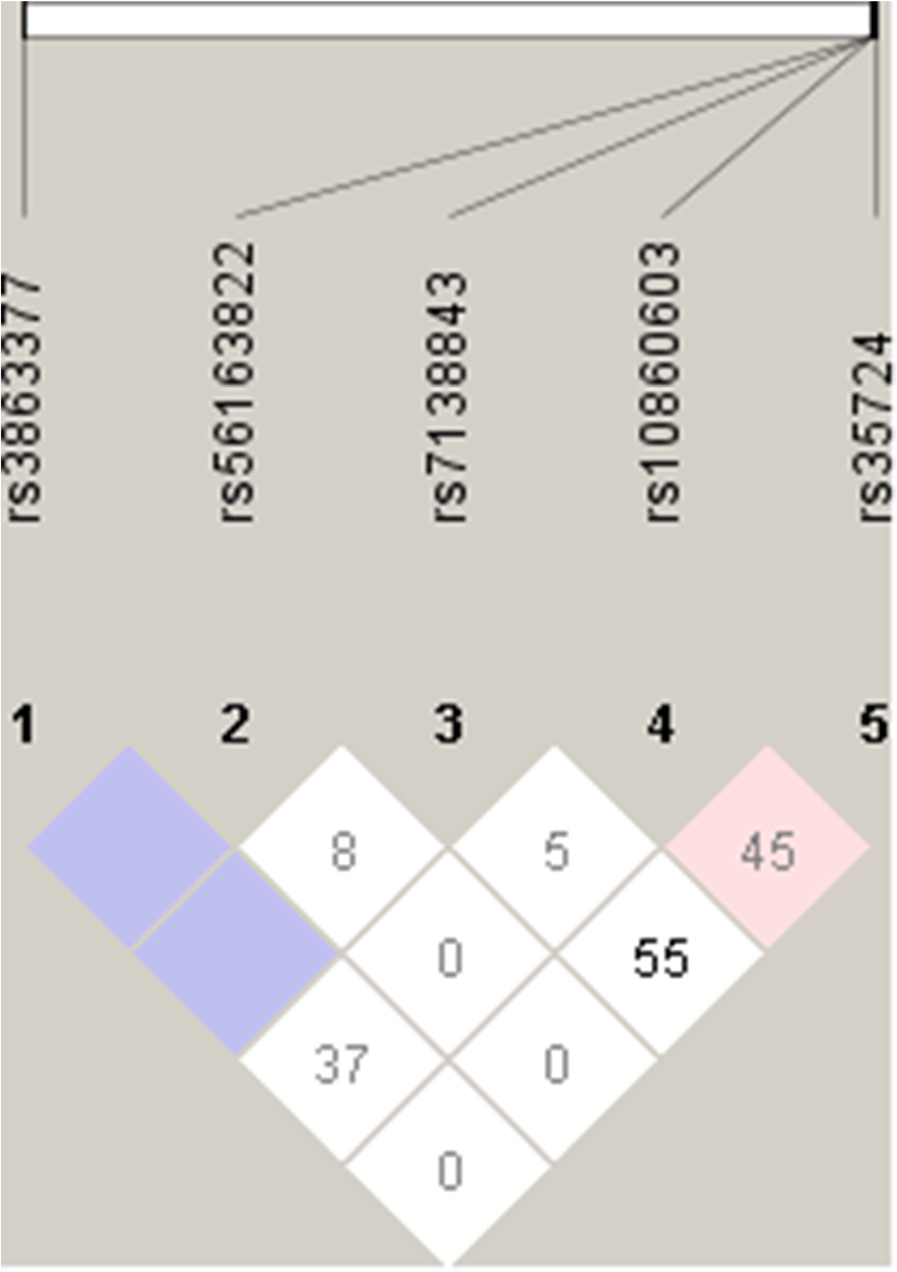
**Pairwise linkage disequilibrium calculations between the five *NR1H4 *SNPs under study in the non-IBD population.** A plot with D’ values; Colour scheme: D’ < 1 and LOD < 2, white, D’ = 1 and LOD < 2, blue, D’ < 1 and LOD ≥ 2, pink.

All individuals, for whom genotype determination could be performed for all five *NR1H4* SNPs under study were included in the haplotype prediction analyses. Four hundred and eighty-six non-IBD cases, along with 243 CD patients and 150 UC patients were thus haplotyped. Twenty haplotypes and up to thirty-nine diplotypes were predicted by the software FAMHAP to exist in the studied cohort. *NR1H4* haplotypes were significantly differentially distributed in the IBD and control groups (Table4, P = 0.003) upon global haplotype distribution analysis. This observation held partially true upon stratification according to disease subtype. Here, the haplotype frequencies differ significantly between the UC patients and the non-IBD control group (Table5, P = 0.004), but not between the CD patients and the control subjects (Table6, P = 0.079). We particularly note that the haplotype 14, *GTTGC*, is predicted to occur significantly (P = 0.005) more frequently in the UC group in comparison with the non-IBD cohort. This haplotype harbours the more frequent allele G at the first SNP position *rs3863377*, which was shown to be significantly associated with IBD even after Bonferroni correction. It is thus a possible risk haplotype for the development of IBD, although we note that the overall frequency for this haplotype is rather low. Upon best reconstruction analysis the significance of the association of the haplotype 14, *GTTGC*, with the UC group was, however, lost (P=0.012) (Table5, italicized section). No significant associations were observed for the predicted diplotype patterns and IBD (data not shown). As shown in the LD plot (Figure1), there was no significant linkage disequilibrium between any of the five *NR1H4* SNPs studied.

**Table 4:** Haplotype analysis of IBD patients and non-IBD controls

**Order**	**Haplotype a) b) c) d) e)**	**Cases (%)**	**Controls (%)**	**OR (C.I.)**	**P**	***Order***	***Haplotype a) b) c) d) e)***	***Cases (%)***	***Controls (%)***	***OR (C.I.)***	***P***
1	G G A G G	405.9 (52.2)	510.1 (52.6)	0.98 (0.81-1.19)		*1*	*G G A G G*	*417 (53.6)*	*530 (54.6)*	*0.96 (0.79-1.16)*	^***a)***^*ns*
2	G G A G C	243.9 (31.3)	261 (26.9)	1.24 (1.01-1.53)	0.064	*2*	*G G A G C*	*235 (30.2)*	*241 (24.7)*	*1.31 (1.06-1.62)*	^***a)***^*0.012*
3	G G A A C	55.9 (7.2)	91.3 (9.4)	0.75 (0.53-1.05)	0.091	*3*	*G G A A C*	*63 (8.1)*	*108 (11.1)*	*0.7 (0.51-0.97)*	^***a)***^*0.03*
4	G G A A G	27.4 (3.5)	47.5 (4.9)	0.71 (0.44-1.14)		*4*	*G G A A G*	*18 (2.3)*	*31 (3.2)*	*0.72 (0.40-1.29)*	^***a)***^*ns*
5	A G A G G	2.7 (0.4)	13.4 (1.4)	0.25 (0.07-0.93)	0.017	*5*	*A G A G G*	*2 (0.3)*	*13 (1.3)*	*0.19 (0.04-0.84)*	*0.017*
6	A G A G C	6.6 (0.8)	12 (1.2)	0.68 (0.26-1.76)		*6*	*A G A G C*	*8 (1.0)*	*14 (1.4)*	*0.71 (0.30-1.70)*	*ns*
7	G G T G C	5 (0.6)	9.1 (0.9)	0.68 (0.23-2.04)		*7*	*G G T G C*	*4 (0.5)*	*9 (0.9)*	*0.55 (0.17-1.80)*	*ns*
8	G T A G G	3.8 (0.5)	7.5 (0.8)	0.63 (0.18-2.17)	NA	*8*	*G T A G G*	*2 (0.3)*	*7 (0.7)*	*0.35 (0.07-1.71)*	*ns*
9	G T A G C	10.4 (1.3)	5.7 (0.6)	2.27 (0.82-6.32)	0.090	*9*	*G T A G C*	*12 (1.5)*	*6 (0.6)*	*2.52 (0.94-6.74)*	*ns*
10	G G T G G	2 (0.3)	3.1 (0.3)	0.79 (0.13-4.70)	NA	*10*	*G G T G G*	*1 (0.1)*	*2 (0.2)*	*0.62 (0.06-6.89)*	*ns*
11	G T A A G	0.8 (0.1)	1.8 (0.2)	NA	NA	*11*	*G T A A G*	*1 (0.1)*	*2 (0.2)*	*0.63 (0.06-6.89)*	*ns*
12	A G A A G	0.2 (0)	2.0 (0.2)	0.13 (0–11.93)	NA	*12*	*A G A A G*	*0*	*1 (0.1)*	*NA*	*ns*
13	G G T A C	2.4 (0.3)	2.3 (0.2)	1.29 (0.21-7.99)	NA	*13*	*G G T A C*	*4 (0.5)*	*4 (0.4)*	*1.25 (0.31-5.00)*	*ns*
14	G T T G C	7.4 (1)	1.0 (0.1)	NA	NA	*14*	*G T T G C*	*7 (0.9)*	*1 (0.1)*	*8.80 (1.08-71.70)*	*0.026*
15	G G T A G	1.1 (0.1)	0.5 (0.1)	NA	NA	*15*	*G G T A G*	*1 (0.1)*	*0*	*NA*	*ns*
16	G T T A G	1.2 (0.2)	1.0 (0.1)	NA	NA	*16*	*G T T A G*	*3 (0.4)*	*1 (0.1)*	*3.75 (0.39-36.15)*	*ns*
17	A G A A C	0.1 (0)	0.6 (0.1)	NA	NA	*17*	*A G A A C*	*0*	*0*	*NA*	*NA*
18	A T A G C	0.4 (0.1)	0 (0)	NA	NA	*18*	*A T A G C*	*0*	*0*	*NA*	*NA*
19	G T T A C	1 (0.1)	0 (0)	NA	NA	*19*	*G T T A C*	*0*	*0*	*NA*	*NA*

389 cases (778 haplotypes) and 485 controls (970 haplotypes) were included in the analysis.

Global haplotype distribution: P = 0.003.

Left table side: Predicted haplotypes obtained with Monte Carlo simulations using FAMHAP. The calculation of odds ratios and a global P value for haplotype distribution was performed on these results using FAMHAP. The haplotype order follows a priority ranking within the control group. Nineteen haplotypes were predicted. The haplotype base positions correspond to a) *rs3863377*, b) *rs56163822*, c) *rs7138843*, d) *rs10860603*, and e) *rs35724*. As FAMHAP tested all haplotypes with a frequency >0.01, the frequency distribution of eight haplotypes (1–7, 9) was included. Relevant P-values for four haplotypes were displayed by FAMHAP. None of the individual haplotypes are significantly differentially distributed between the IBD and control groups.

*OR*, odds ratio; *NA*, not applicable, FAMHAP did not calculate an odds ratio on these results because of a low haplotype frequency value predicted.

Right table side (italics): Most likely occurring haplotype frequencies (haplotype in best reconstruction) predicted by FAMHAP. Based on these predictions significance tests were performed. P-values were calculated using the Fisher’s exact test or (if marked with ^a)^ ) using the Chi-Square test. A Bonferroni-corrected P-value of 0.003 (19 tests) was taken as significance level. According to this, none of the haplotypes appeard to be significantly differently distributed between the case and control groups.

OR, odds ratio; C.I., confidence interval; NA, OR not applicable because of at least one cell count with the value of null; ns, not significant and P >0.05.

**Table 5:** Haplotype analysis of UC patients and non-IBD controls

**Order**	**Haplotype a) b) c) d) e)**	**Cases (%)**	**Controls (%)**	**OR (C.I.)**	**P**	***Order***	***Haplotype a) b) c) d) e)***	***Cases (%)***	***Controls (%)***	***OR (C.I.)***	***P***
1	G G A G G	146.6 (49.5)	507.5 (52.3)	0.89 (0.69-1.16)		*1*	*G G A G G*	*153 (51.7)*	*530 (54.6)*	*0.89 (0.68-1.15)*	^*a)*^*ns*
2	G G A G C	98.8 (33.4)	263.9 (27.2)	1.34 (1.01-1.77)	0.022	*2*	*G G A G C*	*94 (31.8)*	*239 (24.6)*	*1.42 (1.07-1.89)*	^*a)*^*0.015*
3	G G A A C	16.9 (5.7)	89.5 (9.2)	0.59 (0.35-1.02)	0.010	*3*	*G G A A C*	*21 (7.1)*	*111 (11.4)*	*0.59 (0.36-0.96)*	^*a)*^*0.038*
4	G G A A G	14.2 (4.8)	49 (5.1)	0.95 (0.52-1.73)		*4*	*G G A A G*	*9 (3)*	*30 (3.1)*	*0.98 (0.46-2.09)*	^*a)*^*ns*
5	A G A G G	1.2 (0.4)	14.1 (1.5)	0.28 (0.04-1.79)	0.110	*5*	*A G A G G*	*1 (0.3)*	*13 (1.3)*	*0.25 (0.03-1.91)*	*ns*
6	A G A G C	1.7 (0.6)	11 (1.1)	0.5 (0.10-2.53)		*6*	*A G A G C*	*2 (0.7)*	*14 (1.4)*	*0.46 (0.10-2.06)*	*ns*
7	G G T G C	1.6 (0.5)	9.4 (1)	0.54 (0.10-2.97)		*7*	*G G T G C*	*1 (0.3)*	*11 (1.1)*	*0.30 (0.04-2.30)*	*ns*
8	G T A G G	2.2 (0.7)	7.8 (0.8)	0.92 (0.21-4.13)	NA	*8*	*G T A G G*	*1 (0.3)*	*7 (0.7)*	*0.47 (0.06-3.81)*	*ns*
9	G T A G C	2.6 (0.9)	5.4 (0.6)	1.58 (0.36-6.99)	NA	*9*	*G T A G C*	*3 (1)*	*6 (0.6)*	*1.65 (0.41-6.62)*	*ns*
10	G G T G G	0.3 (0.1)	3.0 (0.3)	0.37 (0.01-13.09)	NA	*10*	*G G T G G*	*0*	*2 (0.2)*	*NA*	*ns*
11	G T A A G	0.8 (0.3)	1.8 (0.2)	NA	NA	*11*	*G T A A G*	*1 (0.3)*	*2 (0.2)*	*1.64 (0.15-18.17)*	*ns*
12	A G A A G	0.2 (0.1)	2.0 (0.2)	0.33 (0-30.58)	NA	*12*	*A G A A G*	*0*	*1 (0.1)*	*NA*	*ns*
13	G G T A C	1.1 (0.4)	2.0 (0.2)	1.83 (0.18-18.47)	NA	*13*	*G G T A C*	*2 (0.7)*	*1 (0.1)*	*6.59 (0.60-73.0)*	*ns*
14	G T T G C	4.4 (1.5)	1.0 (0.1)	NA	0.005*	*14*	*G T T G C*	*4 (1.4)*	*1 (0.1)*	*13.27 (1.48-119.30)*	*0.012*
15	G G T A G	1.3 (0.4)	0.7 (0.1)	NA	NA	*15*	*G G T A G*	*1 (0.3)*	*1 (0.1)*	*3.29 (0.20-52.71)*	*ns*
16	G T T A G	1.2 (0.4)	1.0 (0.1)	NA	NA	*16*	*G T T A G*	*2 (0.7)*	*1 (0.1)*	*6.59 (0.60-73.00)*	*ns*
17	A G A A C	0.2 (0.1)	0.9 (0.1)	NA	NA	*17*	*A G A A C*	*0*	*0*	*NA*	*NA*
18	A T A G C	0.7 (0.2)	0	NA	NA	*18*	*A T A G C*	*1 (0.3)*	*0*	*NA*	*ns*
19	G T T A C	0.2 (0.1)	0	NA	NA	*19*	*G T T A C*	*0*	*0*	*NA*	*NA*

**148 cases (296 haplotypes) and controls 485 (970 haplotypes) were included in the analysis**.

**Global haplotype distribution: P=0.004**.

Left table side: Predicted haplotype frequencies obtained with Monte Carlo simulations using FAMHAP: The calculation of odds ratios and a global P-value for haplotype distribution was performed on these results using FAMHAP. The haplotype order follows a priority ranking within the control group. Nineteen haplotypes were predicted. The haplotype base positions correspond to a) *rs3863377*, b) *rs56163822*, c) *rs7138843*, d) *rs10860603*, and e) *rs35724*. As FAMHAP tested all haplotypes with a frequency >0.01, the frequency distribution of eight haplotypes (1-7, 14) was included. Relevant P-values for four haplotypes were displayed by FAMHAP. One single haplotype (14) was significantly differentially distributed between the UC and control groups. OR, odds ratio; NA, not applicable, FAMHAP did not calculate an odds ratio on these results because of a low haplotype frequency value predicted.

Right table side (italics): Most likely occurring haplotype frequencies (haplotypes in best reconstruction) predicted by FAMHAP. Based on these predictions significance tests were performed. P-values were calculated using the Fisher’s exact test or (if marked with ^a)^ ) the Chi-Square-test. A Bonferroni-corrected P-value of 0.003 (19 tests) was taken as significance level. According to this, none of the haplotypes appeared to be significantly different distributed between the UC and control groups. OR, odds ratio; C.I., confidence interval; NA, OR not applicable because of at least one cell count with the value of null; ns, not significant and P > 0.05.

**Table 6:** Haplotype analysis of CD patients and non-IBD controls

**Order**	**Haplotype a) b) c) d) e)**	**Cases (%)**	**Controls (%)**	**OR (C.I.)**	**P**	***Order***	***Haplotype a) b) c) d) e)***	***Cases (%)***	***Controls (%)***	***OR (C.I.)***	***P***
1	G G A G G	258.6 (53.6)	511.2 (52.7)	1.04 (0.83-1.29)		*1*	*G G A G G*	*265 (55.5)*	*530 (55.3)*	*1.01 (0.81-1.26)*	^*a)*^*ns*
2	G G A G C	146.8 (30.5)	259.0 (26.7)	1.20 (0.95-1.53)		*2*	*G G A G C*	*140 (29.4)*	*239 (24.9)*	*1.25 (0.98-1.60)*	^*a)*^*ns*
3	G G A A C	39.3 (8.2)	93.9 (9.7)	0.83 (0.56-1.22)		*3*	*G G A A C*	*44 (9.2)*	*110 (11.5)*	*0.79 (0.54-1.14)*	^*a)*^*ns*
4	G G A A G	13 (2.7)	45.9 (4.7)	0.56 (0.30-1.04)	0.062	*4*	*G G A A G*	*9 (1.9)*	*31 (3.2)*	*0.58 (0.27-1.22)*	*ns*
5	A G A G G	2.1 (0.4)	14.4 (1.5)	0.29 (0.07-1.24)	0.053	*5*	*A G A G G*	*1 (0.2)*	*13 (1.4)*	*0.15 (0.02-1.17)*	*0.044*
6	A G A G C	3.9 (0.8)	11.6 (1.2)	0.68 (0.22-2.15)		*6*	*A G A G C*	*5 (1)*	*14 (1.5)*	*0.72 (0.26-2.00)*	*ns*
7	G G T G C	3.8 (0.8)	9.4 (1.0)	0.81 (0.24-2.68)		*7*	*G G T G C*	*5*	*11*	*0.91 (0.32-2.65)*	*ns*
8	G T A G G	2.1 (0.4)	7.8 (0.8)	0.53 (0.11-2.49)	NA	*8*	*G T A G G*	*1 (0.2)*	*7 (0.7)*	*0.29 (0.04-2.33)*	*ns*
9	G T A G C	6.1 (1.3)	5.7 (0.6)	2.19 (0.70-6.91)	0.122	*9*	*G T A G C*	*7 (1.5)*	*6 (0.6)*	*2.37 (0.79-7.09)*	*ns*
10	G G T G G	1.5 (0.3)	3.1 (0.3)	0.98 (0.14-6.86)	NA	*10*	*G G T G G*	*1 (0.2)*	*2 (0.2)*	*1.01 (0.09-11.13)*	*ns*
11	G T A A G	0	1.6 (0.2)	NA	NA	*11*	*G T A A G*	*0*	*2 (0.2)*	*NA*	*ns*
12	A G A A G	0	2.1 (0.2)	NA	NA	*12*	*A G A A G*	*0*	*1 (0.1)*	*NA*	*ns*
13	G G T A C	0.9 (0.2)	2.5 (0.3)	0.73 (0.07-7.9)	NA	*13*	*G G T A C*	*0*	*2 (0.2)*	*NA*	*ns*
14	G T T G C	3.1 (0.6)	0.9 (0.1)	NA	NA	*14*	*G T T G C*	*3 (0.6)*	*1 (0.1)*	*6.07 (0.63-58.53)*	*ns*
15	G G T A G	NA	NA	NA	NA	*15*	*G G T A G*	*0*	*0*	*NA*	*NA*
16	G T T A G	0.7 (0.1)	1.0 (0.1)	NA	NA	*16*	*G T T A G*	*1 (0.2)*	*1 (0.1)*	*2.02 (0.13-32.30)*	*ns*
17	A G A A C	NA	NA	NA	NA	*17*	*A G A A C*	*0*	*0*	*NA*	*NA*
18	A T A G C	NA	NA	NA	NA	*18*	*A T A G C*	*0*	*0*	*NA*	*NA*
19	G T T A C	NA	NA	NA	NA	*19*	*G T T A C*	*0*	*0*	*NA*	*NA*

241 cases (482 haplotypes) and 485 controls (970 haplotypes) were included in the analysis.

Global haplotype distribution: P=0.079.

Left table side: Predicted haplotype frequencies obtained with Monte Carlo simulations using FAMHAP: The calculation of odds ratios and a global P-value for haplotype distribution was performed on these results using FAMHAP. The haplotype order follows a priority ranking within the control group. Nineteen haplotypes were predicted. The haplotype base positions correspond to a) *rs3863377*, b) *rs56163822*, c) *rs7138843*, d) *rs10860603*, and e) *rs35724*. As FAMHAP tested all haplotypes with a frequency >0.01, the frequency distribution of eight haplotypes (1-7, 9) was included. Relevant P-values for three haplotypes were displayed by FAMHAP. None of the individual haplotypes are significantly differentially distributed between the CD and control groups. OR, odds ratio; NA, not applicable, FAMHAP did not calculate an odds ratio on these results because of a low haplotype frequency value predicted.

Right table side (italics): Most likely occurring haplotype frequencies (haplotypes in best reconstruction) predicted by FAMHAP. Based on these predictions significance tests were performed.

P-values were calculated using the Fisher’s exact test or (if marked with ^a)^ ) the Chi-Square-test. A Bonferroni-corrected P-value of 0.003 (19 tests) was taken as significance level. According to this, none of the haplotypes appeared to be significantly different distributed between the CD and control groups.

OR, odds ratio; C.I., confidence interval; NA, OR not applicable because of at least one cell count with the value of null; ns, not significant and P > 0.05.

## Discussion

The complex pathophysiology of IBD still remains largely unelucidated, although multiple factors, both genetic and environmental, are clearly involved. SNPs and mutations within several genes have been proposed to be associated with the risk to develop IBD. Prior studies have revealed more than 70 genes that are potentially associated with IBD [34,35]. The region on chromosome *16q11-12* named *IBD1* was identified in 1996, and the fine mapping of this region led to the identification of the *NOD2* (nucleotide-binding oligomerization domain 2)/*CARD15* (caspase activation recruitment domain 15) genes [36,37], and a member of a family of pattern recognition receptors (PRRs) that recognizes microbial components and modifies inflammatory responses to bacterial triggers such as lipopolysaccharides (LPS), through the activation of NF-κB [38,39]. Furthermore, genes that play roles in immunological cell-cell interactions and signaling, such as the tumor necrosis factor receptor 1 (*TNFR1*) [40], the interleukin-23 receptor (*IL23R*) [41], and other genes that are involved in immune response to bacteria, such as the toll-like receptor 4 (*TLR4*) [42,43], have been proposed to be associated with IBD. In addition, regulatory genes, such as the protein tyrosine phosphatase N2 (*PTPN2*) [44,45] and the anti-inflammatory nuclear receptor peroxisome proliferator-activated receptor-*γ* (*PPARγ*) [46], as well as genes encoding membrane transporters multidrug resistance gene 1 (*MDR1)*[47-49] and the organic cation transporter 1/2 (*OCTN1/2*) [47,50] have been proposed to be associated with the risk of chronic mucosal inflammation.

In this report, we describe the identification of single nucleotide polymorphisms associated with the diagnosis of IBD within the *NR1H4* gene, encoding the nuclear receptor for bile acids, FXR, in a well-sized European cohort. Five *NR1H4* SNPs were analyzed, all of which have previously been studied in the context of other human disease conditions: *rs3863377*, *rs7138843*, *rs56163822*, *rs35724*, and *rs10860603*. The *NR1H4* variants *rs7138843* and *rs56163822* have been previously shown to be inversely associated with cholelithiasis in a Mexican population and may thus play a protective role in gallstone disease, while the variant *rs3863377* showed no association with cholelithiasis [51]. The variant *rs56163822* was found to be more common in a British control group than in patients with intrahepatic cholestasis of pregnancy (ICP), although this difference did not reach statistical significance [32]. *NR1H4* variants *rs35724* and *rs10860603* have been previously shown to be significantly associated with elevated body mass index and obesity [52]. IBD is often associated with hepatobiliary manifestations, [53,54] implying that the etiology of the diseases affecting the two organs, intestine and liver, may have common factors, also supported by our findings that the same *NR1H4* genetic variants may be associated with both.

The variant *rs3863377* is located in the 5’ region of the *NR1H4* gene, whereas the *rs7138843* lies within the *NR1H4* intron 7 and variants *rs35724* and *rs10860603* within *NR1H4* intron 9. In none of these cases is it known, how the presence of the SNP may affect the expression and/or molecular function of FXR. As the SNP *rs3863377* is located within the 5’ region, it may alter a binding site for a transcription factor and may thus affect *NR1H4* gene expression. The intronic SNPs *rs7138843*, *rs10860603*, and *rs35724* could potentially influence splicing of the FXR mRNA. The substitution *-1 G > T* in *rs56163822* lies in the base position adjacent to the translation initiation site, and was shown to lead to reduced FXR protein expression and decreased level of FXR-dependent promoter activation in human embryonic kidney cells. In another study the functional activity of the *-1 G > T* variant also appeared to be compromised, although transcriptional and translational efficiencies of the variant appeared comparable to the wild-type in cell-free assays and in HeLa cells [55]. Interestingly, the mRNA expression levels of the FXR target genes *SHP* and *OATP1B3* are significantly reduced in the livers of the carriers of the *rs56163822* allele, while the FXR mRNA expression level remains comparable, further indicating that this polymorphism may rather lead to weakened function than to reduced expression level of FXR.

In our current genotyping analysis we have found that for the *NR1H4* variant *rs3863377*, the IBD population has a significantly lower frequency of carriers of the rarer allele than the healthy population, suggesting that this 5’ region SNP may confer a protective effect against the disease. In the case of the *rs56163822 NR1H4* variant, the rare allele is significantly more prevalent in the IBD population, suggesting that previously reported reduced FXR function exhibited by this variant may contribute to IBD pathogenesis. In the case of the rare *NR1H4* variant under study, *rs7138843*, and the common SNPs *rs10860603* and *rs35724*, no significant differences between the study populations were observed. In agreement with the associations observed for two of the five single SNP variants, the predicted global haplotype pattern was significantly different in IBD patients and non-IBD controls.

In our study, five *NR1H4* SNPs were investigated. During the preparation of our manuscript, Nijmeijer *et al.*[56] published a study showing that mRNA expression of FXR and its target gene *SHP* are decreased in the ileum of Crohn’s disease patients, in further support of the importance of the role for FXR in IBD. These authors also studied potential association of nine *NR1H4* SNPs with IBD in a Dutch population, but did not discover any associations that remained significant upon correction for multiple testing. We note that in their analysis Nijmeijer *et al.* did not include the SNP *rs3863377*, the inverse association by which with IBD remained significant even after Bonferroni correction in the current study. As numerous further polymorphisms are known to exist in the *NR1H4* gene (http://www.ncbi.nlm.nih.gov/snp/), our report, as well as that by Nijmeijer *et al.*, serve as initial characterizations of the role of FXR genetic variants in IBD. Furthermore, these findings warrant further studies into genetic variants in the *NR1H4* gene in the context of other inflammatory conditions affecting further tissues that express FXR.

FXR ligands, such as the hydrophilic bile acid ursodeoxycholic acid, have been proposed as attractive options for the therapy of liver diseases, such as cholestatic disease and non-alcoholic fatty liver disease [57]. Our finding that FXR genetic variants are associated with IBD, together with prior observations on FXR expression being altered in Crohn’s disease [56] and on FXR promoting intestinal barrier integrity [27] and antibacterial defence [26], further emphasizes the potential benefits of FXR ligand administration also in IBD. We further speculate that testing for genetic variation in the *NR1H4* gene may contribute to the early IBD diagnosis and prediction of therapy response in the future.

## Conclusions

In conclusion, our results further support the role for FXR as a modulator of intestinal inflammation and as an important player in enteroprotection. The link between the bile acid receptor FXR and IBD also further emphasizes the potential importance of bile acid homeostasis and metabolism in the pathogenesis of IBD.

## Methods

### Study subjects

The study population was European, and comprised of 591 healthy subjects and 547 IBD patients, from which 203 were diagnosed to suffer from UC and 334 from CD. The IBD subjects were recruited at the centers participating in the Swiss Inflammatory Bowel Disease Cohort Study (SIBDCS) [58]. For the IBD patients, the diagnosis of UC or CD was confirmed by the study investigators based on clinical presentation, endoscopic findings, and histology. Non-IBD controls were recruited from gastroenterological patients undergoing surveillance colonoscopy, and showed no symptoms of IBD. History of colorectal cancer was used as an exclusion criterion for both IBD patients and non-IBD controls. All subjects provided their written informed consent to be included in the study. Ethical approvals were obtained from the local medical ethical committees of all study sites involved in the study: 1) The Swiss IBD Cohort Study (SIBDCS) (the participating centers are listed in http://ibdcohort.ch/index.php?id = 94&L = 2; ethical license EK-1316). 2) The Bioethical Committee at the Maria Sklodowska-Curie Memorial Cancer Centre and Institute of Oncology, Warsaw, Poland (ethical licenses 25/2006 and 25/2006/2007).

### DNA extraction

Genomic DNAs were extracted from either EDTA-blood or intestinal biopsies using the QIAamp DNA Mini Kit (QIAGEN, Hombrechtikon, Switzerland) or the TRIzol reagent (Invitrogen, Basel, Switzerland), respectively, according to the manufacturer’s instructions. The genomic DNAs were quantified with a NanoDrop ND-1000 spectrophotometer (NanoDrop Technologies, Wilmington, DE) and diluted to a final concentration of 10 ng/μl.

### Genotyping of *NR1H4* single nucleotide polymorphisms

Genotyping of the five *NR1H4* SNPs was performed using TaqMan allelic discrimination assays. The cycling was performed on an 7900HT Fast Real-Time PCR system (Applied Biosystems, Rotkreuz, Switzerland) by using the inventoried TaqMan SNP Genotyping Assays C_28000279_10, C_25598395, C_25598386_10, C_2366616_10, C_2800610_10 for the SNPs *rs3863377*, *rs7138843*, *rs56163822*, *rs35724*, and *rs1086060*, respectively. Twenty nanograms of each genomic DNA was used per PCR reaction in a volume of 5 μl. The amplification run conditions were: Once 50°C for 2 min, once 95°C for 10 min, 45 times 95°C for 15 sec, and 60°C for 1 min.

### Statistical analysis

Statistical analysis for the individual SNP associations was performed using the software package SPSS 18 (SPSS Inc., Chicago, IL). The Chi-square test or Fisher’s exact test were used to determine associations between individual SNPs and subject phenotypes. A P-value of <0.05 was considered as significant in non-corrected statistical tests and of <0.01 after correction for multiple testing for the five SNPs (according to Bonferroni). The software package PSPower (http://biostat.mc.vanderbilt.edu/twiki/bin/view/Main/PowerSampleSize) was used for retrospective power calculations. A retrospective power analysis of the applied statistical tests on genotype distributions revealed a power of 0.525 for the SNP *rs3863377* and a power of 0.323 for the SNP *rs56163822*, when considering a Bonferroni-corrected alpha level of 0.01 and the detected ORs as shown in Table2 for the applied *χ*^2^-tests. Linkage disequilibria (LD) were calculated using D’ statistics and the software package Haploview (http://www.haploview.com). Haplotype predictions and frequency estimations were performed using the software tool FAMHAP (http://www.famhap.meb.uni-bonn.de). FAMHAP performs a permutation test on associations between estimated haplotypes and the affection state based on Monte Carlo simulations. The expectation maximization (EM) algorithm was used to obtain maximum-likelihood estimates of the haplotype frequencies of the sample composed of cases and controls. Individuals with several possible haplotype explanations are assigned with a likelihood weight to each possible haplotype and its calculated frequency estimate. A contingency table is constructed summing up all individuals’ weighted haplotype explanations for each haplotype and the chi-square statistics computed. The corresponding P-value is assessed via Monte Carlo simulation, i.e. in each replication of the algorithm a sample composed of a subgroup of case and control samples is randomly drawn and permuted. FAMHAP implements the calculation of the global P-value via Monte Carlo simulations, as the cell counts used in the contingency table are based on haplotype frequency estimates with increased variances, not on real haplotype counts, which, as a result, does not necessarily follow exactly a chi-square distribution [59,60]. A value of P<0.05 was considered to be significant. Bonferroni-corrected P-values (P<0.006, corrected for eight haplotypes that FAMHAP considered to be relevant to test for) were defined as the significance level for single haplotype comparisons in the white sections of Tables 4-6. In addition, haplotypes in best reconstruction (not weighted) were listed for the case and control groups in Tables 4-6 (grey sections) and used for association analysis performing Fisher’s exact tests or, in case of high cell counts (11 or more), Chi-square tests. Bonferroni-corrected significance levels (p<0.003, corrected for 19 haplotypes) were used for significance testing.

### Endnotes

^a^Members of the Swiss Inflammatory Bowel Disease Cohort Study (SIBDCS) group: Pierluigi Ballabeni, Peter Bauerfeind, Christoph Beglinger, Stefan Begré, José Bengoa, Janek Binek, Daniel Boller, Jan Borovicka, Christian Braegger, Patrick Brun, Patrick Bühr, Bernard Burnand, Rafael Camara, Dominique Criblez, Philippe de Saussure, Lukas Degen, Joakim Delarive, Tobias Ehmann, Matthias Engelmann, Ali El Wafa, Christian Felley, Alain Frei, Remus Frei, Michael Fried, Florian Froehlich, Suzanne Gallot-Lavallée, Tilman Gerlach, Martin Geyer, Marc Girardin, Oliver Goetze, Horst Haack, Serge Hediger, Peter Hengstler, Klaas Heyland, Patrick Janiak, Pascal Juillerat, Vera Kessler Brondolo, Christoph Knoblauch, Gerd A. Kullak-Ublick, Michael Manz, Rémy Meier, Christa Meyenberger, Pierre Michetti, Christian Mottet, Christoph Müller, Beat Müllhaupt, Thierry Nicolet, Andreas Nydegger, Isabelle Pache, Franziska Piccoli, Julia Pilz, Valérie Pittet, Ronald Rentsch, Jean-Pierre Rey, Silvia Rihs, Daniela Rogler, Gerhard Rogler, Markus Sagmeister, Bernhard Sauter, Niklaus Schaub, Susanne Schibli, Alain Schoepfer, Franck Seibold, Johannes Spalinger, Philippe Stadler, Michael Steuerwald, Alex Straumann, Michael Sulz, Michela Schäppi, Joël Thorens, John-Paul Vader, Stephan Vavricka, Jürg Vögtlin, Roland Von Känel, Gert Wachter, Jürg Wermuth, Paul Wiesel.

## Abbreviations

IBD: Inflammatory bowel disease; UC: Ulcerative colitis; CD: Crohn’s disease; FXR: Farnesoid X receptor; SNP: Single nucleotide polymorphism; *NR1H4*: Nuclear receptor subfamily 1, group H, member 4; OR: Odds ratio; CI: Confidence interval; MAF: Minor allele frequency.

## Competing interests

The authors declare that they have no competing interests.

## Authors’ contributions

RA performed the genotyping, interpreted data, and wrote the first draft of the manuscript. JM performed the statistical analysis of the data. KT, JR, and PG performed the sample collection and management. GK and GR contributed to the supervision and design of the study. JJE conceived and supervised the study, and contributed to the data interpretation and writing of the manuscript. All authors read and approved of the final manuscript.

## Author details

^1^Department of Clinical Pharmacology and Toxicology, University Hospital, Zurich, Switzerland. ^2^Department of Biomedicine, Institute of Biochemistry and Genetics, University of Basel, Switzerland and Division of Gastroenterology, Regional Hospital, Langenthal, Switzerland. ^3^Department of Gastroenterology and Hepatology, Medical Center for Postgraduate Education and the Maria Skłodowska Curie Memorial Cancer Center, Institute of Oncology, Warsaw, Poland. ^4^Division of Gastroenterology and Hepatology, University Hospital, Zurich, Switzerland.

## References

[B1] Kaser A Zeissig S Blumberg RS Inflammatory bowel disease Annu Rev Immunol 2010 28 573 621 10.1146/annurev-immunol-030409-101225 20192811 PMC4620040

[B2] Jess T Riis L Jespersgaard C Hougs L Andersen PS Orholm MK Binder V Munkholm P Disease concordance, zygosity, and NOD2/CARD15 status: follow-up of a population-based cohort of Danish twins with inflammatory bowel disease Am J Gastroenterol 2005 100 2486 2492 10.1111/j.1572-0241.2005.00224.x 16279904

[B3] Chawla A Repa JJ Evans RM Mangelsdorf DJ Nuclear receptors and lipid physiology: opening the X-files Science 2001 294 1866 1870 10.1126/science.294.5548.1866 11729302

[B4] Mangelsdorf DJ Thummel C Beato M Herrlich P Schutz G Umesono K Blumberg B Kastner P Mark M Chambon P The nuclear receptor superfamily: the second decade Cell 1995 83 835 839 10.1016/0092-8674(95)90199-X 8521507 PMC6159888

[B5] Glass CK Differential recognition of target genes by nuclear receptor monomers, dimers, and heterodimers Endocr Rev 1994 15 391 407 8076589 10.1210/edrv-15-3-391

[B6] Eloranta JJ Kullak-Ublick GA Coordinate transcriptional regulation of bile acid homeostasis and drug metabolism Arch Biochem Biophys 2005 433 397 412 10.1016/j.abb.2004.09.019 15581596

[B7] Wang H Chen J Hollister K Sowers LC Forman BM Endogenous bile acids are ligands for the nuclear receptor FXR/BAR Mol Cell 1999 3 543 553 10360171 10.1016/s1097-2765(00)80348-2

[B8] Parks DJ Blanchard SG Bledsoe RK Chandra G Consler TG Kliewer SA Stimmel JB Willson TM Zavacki AM Moore DD Bile acids: natural ligands for an orphan nuclear receptor Science 1999 284 1365 1368 10.1126/science.284.5418.1365 10334993

[B9] Makishima M Okamoto AY Repa JJ Tu H Learned RM Luk A Hull MV Lustig KD Mangelsdorf DJ Shan B Identification of a nuclear receptor for bile acids Science 1999 284 1362 1365 10.1126/science.284.5418.1362 10334992

[B10] Huber RM Murphy K Miao B Link JR Cunningham MR Rupar MJ Gunyuzlu PL Haws TF Kassam A Powell F Generation of multiple farnesoid-X-receptor isoforms through the use of alternative promoters Gene 2002 290 35 43 10.1016/S0378-1119(02)00557-7 12062799

[B11] Anisfeld AM Kast-Woelbern HR Meyer ME Jones SA Zhang Y Williams KJ Willson T Edwards PA Syndecan-1 expression is regulated in an isoform-specific manner by the farnesoid-X receptor J Biol Chem 2003 278 20420 20428 10.1074/jbc.M302505200 12660231

[B12] Laffitte BA Kast HR Nguyen CM Zavacki AM Moore DD Edwards PA Identification of the DNA binding specificity and potential target genes for the farnesoid X-activated receptor J Biol Chem 2000 275 10638 10647 10.1074/jbc.275.14.10638 10744760

[B13] Eloranta JJ Kullak-Ublick GA The role of FXR in disorders of bile acid homeostasis Physiology (Bethesda) 2008 23 286 295 10.1152/physiol.00020.2008 18927204

[B14] Lee H Zhang Y Lee FY Nelson SF Gonzalez FJ Edwards PA FXR regulates organic solute transporters alpha and beta in the adrenal gland, kidney, and intestine J Lipid Res 2006 47 201 214 16251721 10.1194/jlr.M500417-JLR200

[B15] Landrier JF Eloranta JJ Vavricka SR Kullak-Ublick GA The nuclear receptor for bile acids, FXR, transactivates human organic solute transporter-alpha and -beta genes Am J Physiol Gastrointest Liver Physiol 2006 290 G476 G485 10.1152/ajpgi.00430.2005 16269519

[B16] Plass JR Mol O Heegsma J Geuken M Faber KN Jansen PL Muller M Farnesoid X receptor and bile salts are involved in transcriptional regulation of the gene encoding the human bile salt export pump Hepatology 2002 35 589 596 10.1053/jhep.2002.31724 11870371

[B17] Ananthanarayanan M Balasubramanian N Makishima M Mangelsdorf DJ Suchy FJ Human bile salt export pump promoter is transactivated by the farnesoid X receptor/bile acid receptor J Biol Chem 2001 276 28857 28865 10.1074/jbc.M011610200 11387316

[B18] Schuetz EG Strom S Yasuda K Lecureur V Assem M Brimer C Lamba J Kim RB Ramachandran V Komoroski BJ Disrupted bile acid homeostasis reveals an unexpected interaction among nuclear hormone receptors, transporters, and cytochrome P450 J Biol Chem 2001 276 39411 39418 10.1074/jbc.M106340200 11509573

[B19] Neimark E Chen F Li X Shneider BL Bile acid-induced negative feedback regulation of the human ileal bile acid transporter Hepatology 2004 40 149 156 15239098 10.1002/hep.20295

[B20] Zollner G Wagner M Fickert P Geier A Fuchsbichler A Silbert D Gumhold J Zatloukal K Kaser A Tilg H Role of nuclear receptors and hepatocyte-enriched transcription factors for Ntcp repression in biliary obstruction in mouse liver Am J Physiol Gastrointest Liver Physiol 2005 289 G798 G805 10.1152/ajpgi.00319.2004 16002565

[B21] Eloranta JJ Jung D Kullak-Ublick GA The human Na + −taurocholate cotransporting polypeptide gene is activated by glucocorticoid receptor and peroxisome proliferator-activated receptor-gamma coactivator-1alpha, and suppressed by bile acids via a small heterodimer partner-dependent mechanism Mol Endocrinol 2006 20 65 79 16123152 10.1210/me.2005-0159

[B22] Chiang JY Kimmel R Weinberger C Stroup D Farnesoid X receptor responds to bile acids and represses cholesterol 7alpha-hydroxylase gene (CYP7A1) transcription J Biol Chem 2000 275 10918 10924 10.1074/jbc.275.15.10918 10753890

[B23] Goodwin B Jones SA Price RR Watson MA McKee DD Moore LB Galardi C Wilson JG Lewis MC Roth ME A regulatory cascade of the nuclear receptors FXR, SHP-1, and LRH-1 represses bile acid biosynthesis Mol Cell 2000 6 517 526 10.1016/S1097-2765(00)00051-4 11030332

[B24] Wildenberg ME van den Brink GR FXR activation inhibits inflammation and preserves the intestinal barrier in IBD Gut 2011 60 432 433 10.1136/gut.2010.233304 21270116

[B25] Vavassori P Mencarelli A Renga B Distrutti E Fiorucci S The bile acid receptor FXR is a modulator of intestinal innate immunity J Immunol 2009 183 6251 6261 10.4049/jimmunol.0803978 19864602

[B26] Inagaki T Moschetta A Lee YK Peng L Zhao G Downes M Yu RT Shelton JM Richardson JA Repa JJ Regulation of antibacterial defense in the small intestine by the nuclear bile acid receptor Proc Natl Acad Sci U S A 2006 103 3920 3925 10.1073/pnas.0509592103 16473946 PMC1450165

[B27] Gadaleta RM van Erpecum KJ Oldenburg B Willemsen EC Renooij W Murzilli S Klomp LW Siersema PD Schipper ME Danese S Farnesoid X receptor activation inhibits inflammation and preserves the intestinal barrier in inflammatory bowel disease Gut 2011 60 463 472 10.1136/gut.2010.212159 21242261

[B28] Gadaleta RM Oldenburg B Willemsen ECL Spit M Murzilli S Salvatore L Klomp LWJ Siersema PD van Erpecum KJ van Mil SWC Activation of bile salt nuclear receptor FXR is repressed by pro-inflammatory cytokines activating NF-[kappa]B signaling in the intestine. *Biochimica et Biophysica Acta (BBA)* Molecular Basis of Disease 2011 1812 851 858 10.1016/j.bbadis.2011.04.005 21540105

[B29] Lian F Xing X Yuan G Schafer C Rauser S Walch A Rocken C Ebeling M Wright MB Schmid RM Farnesoid X receptor protects human and murine gastric epithelial cells against inflammation-induced damage Biochem J 2011 438 315 323 10.1042/BJ20102096 21619550

[B30] Kovacs P Kress R Rocha J Kurtz U Miquel JF Nervi F Méndez-Sánchez N Uribe M Bock HH Schirin-Sokhan R Variation of the gene encoding the nuclear bile salt receptor FXR and gallstone susceptibility in mice and humans J Hepatol 2008 48 116 124 10.1016/j.jhep.2007.07.027 17931734

[B31] Wadsworth CA Dixon PH Wong JH Chapman MH McKay SC Sharif A Spalding DR Pereira SP Thomas HC Taylor-Robinson SD Genetic factors in the pathogenesis of cholangiocarcinoma Dig Dis 2011 29 93 97 10.1159/000324688 21691113 PMC3696362

[B32] Van Mil SW Milona A Dixon PH Mullenbach R Geenes VL Chambers J Shevchuk V Moore GE Lammert F Glantz AG Functional variants of the central bile acid sensor FXR identified in intrahepatic cholestasis of pregnancy Gastroenterology 2007 133 507 516 10.1053/j.gastro.2007.05.015 17681172

[B33] Chen X-Q Wang L-L Shan Q-W Tang Q Deng Y-N Lian S-J Yun X A novel heterozygous *NRIH4* termination codon mutation in idiopathic infantile cholestasis World Journal of Pediatrics 2012 8 67 71 10.1007/s12519-011-0299-z 21633855

[B34] Zhang H Massey D Tremelling M Parkes M Genetics of inflammatory bowel disease: clues to pathogenesis Br Med Bull 2008 87 17 30 10.1093/bmb/ldn031 18753178

[B35] Franke A McGovern DP Barrett JC Wang K Radford-Smith GL Ahmad T Lees CW Balschun T Lee J Roberts R Genome-wide meta-analysis increases to 71 the number of confirmed Crohn’s disease susceptibility loci Nat Genet 2010 42 1118 1125 10.1038/ng.717 21102463 PMC3299551

[B36] Hugot J-P Chamaillard M Zouali H Lesage S Cezard J-P Belaiche J Almer S Tysk C O’Morain CA Gassull M Association of NOD2 leucine-rich repeat variants with susceptibility to Crohn’s disease Nature 2001 411 599 603 10.1038/35079107 11385576

[B37] Ogura Y Bonen DK Inohara N Nicolae DL Chen FF Ramos R Britton H Moran T Karaliuskas R Duerr RH A frameshift mutation in NOD2 associated with susceptibility to Crohn’s disease Nature 2001 411 603 606 10.1038/35079114 11385577

[B38] Inohara N Koseki T Lin J del Peso L Lucas PC Chen FF Ogura Y Nunez G An induced proximity model for NF-kappa B activation in the Nod1/RICK and RIP signaling pathways J Biol Chem 2000 275 27823 27831 10880512 10.1074/jbc.M003415200

[B39] Inohara N Ogura Y Chen FF Muto A Nunez G Human Nod1 confers responsiveness to bacterial lipopolysaccharides J Biol Chem 2001 276 2551 2554 10.1074/jbc.M009728200 11058605

[B40] Sashio H Tamura K Ito R Yamamoto Y Bamba H Kosaka T Fukui S Sawada K Fukuda Y Satomi M Polymorphisms of the TNF gene and the TNF receptor superfamily member 1B gene are associated with susceptibility to ulcerative colitis and Crohn’s disease, respectively Immunogenetics 2002 53 1020 1027 10.1007/s00251-001-0423-7 11904678

[B41] Fisher SA Tremelling M Anderson CA Gwilliam R Bumpstead S Prescott NJ Nimmo ER Massey D Berzuini C Johnson C Genetic determinants of ulcerative colitis include the ECM1 locus and five loci implicated in Crohn’s disease Nat Genet 2008 40 710 712 10.1038/ng.145 18438406 PMC2719289

[B42] Oostenbrug LE Drenth JP de Jong DJ Nolte IM Oosterom E van Dullemen HM van der Linde K te Meerman GJ van der Steege G Kleibeuker JH Association between Toll-like receptor 4 and inflammatory bowel disease Inflamm Bowel Dis 2005 11 567 575 15905704 10.1097/01.mib.0000161305.81198.0f

[B43] Franchimont D Vermeire S El Housni H Pierik M Van Steen K Gustot T Quertinmont E Abramowicz M Van Gossum A Deviere J Deficient host-bacteria interactions in inflammatory bowel disease? The toll-like receptor (TLR)-4 Asp299gly polymorphism is associated with Crohn’s disease and ulcerative colitis Gut 2004 53 987 992 10.1136/gut.2003.030205 15194649 PMC1774122

[B44] Wellcome Trust Case Control Consortium Genome-wide association study of 14,000 cases of seven common diseases and 3,000 shared controls Nature 2007 447 661 678 10.1038/nature05911 17554300 PMC2719288

[B45] Franke A Balschun T Karlsen TH Hedderich J May S Lu T Schuldt D Nikolaus S Rosenstiel P Krawczak M Replication of signals from recent studies of Crohn’s disease identifies previously unknown disease loci for ulcerative colitis Nat Genet 2008 40 713 715 10.1038/ng.148 18438405

[B46] Sugawara K Olson TS Moskaluk CA Stevens BK Hoang S Kozaiwa K Cominelli F Ley KF McDuffie M Linkage to peroxisome proliferator-activated receptor-[gamma] in SAMP1/YitFc mice and in human Crohn’s disease Gastroenterology 2005 128 351 360 10.1053/j.gastro.2004.11.001 15685547

[B47] Waller S Tremelling M Bredin F Godfrey L Howson J Parkes M Evidence for association of OCTN genes and IBD5 with ulcerative colitis Gut 2006 55 809 814 10.1136/gut.2005.084574 16361305 PMC1856215

[B48] Schwab M Schaeffeler E Marx C Fromm MF Kaskas B Metzler J Stange E Herfarth H Schoelmerich J Gregor M Association between the C3435T MDR1 gene polymorphism and susceptibility for ulcerative colitis Gastroenterology 2003 124 26 33 10.1053/gast.2003.50010 12512026

[B49] Brant SR Panhuysen CIM Nicolae D Reddy DM Bonen DK Karaliukas R Zhang L Swanson E Datta LW Moran T MDR1 Ala893 Polymorphism Is Associated with Inflammatory Bowel Disease Am J Hum Genet 2003 73 1282 1292 10.1086/379927 14610718 PMC1180394

[B50] Peltekova VD Wintle RF Rubin LA Amos CI Huang Q Gu X Newman B Van Oene M Cescon D Greenberg G Functional variants of OCTN cation transporter genes are associated with Crohn disease Nat Genet 2004 36 471 475 10.1038/ng1339 15107849

[B51] Kovacs P Kress R Rocha J Kurtz U Miquel JF Nervi F Mendez-Sanchez N Uribe M Bock HH Schirin-Sokhan R Variation of the gene encoding the nuclear bile salt receptor FXR and gallstone susceptibility in mice and humans J Hepatol 2008 48 116 124 10.1016/j.jhep.2007.07.027 17931734

[B52] van den Berg SW Dolle ME Imholz S van der AD van t Slot R Wijmenga C Verschuren WM Strien C Siezen CL Hoebee B Genetic variations in regulatory pathways of fatty acid and glucose metabolism are associated with obesity phenotypes: a population-based cohort study Int J Obes (Lond) 2009 33 1143 1152 10.1038/ijo.2009.152 19652658

[B53] Christophi C Hughes ER Hepatobiliary disorders in inflammatory bowel disease Surg Gynecol Obstet 1985 160 187 193 3881836

[B54] Navaneethan U Shen B Hepatopancreatobiliary manifestations and complications associated with inflammatory bowel disease Inflamm Bowel Dis 2010 16 1598 1619 10.1002/ibd.21219 20198712

[B55] Marzolini C Tirona RG Gervasini G Poonkuzhali B Assem M Lee W Leake BF Schuetz JD Schuetz EG Kim RB A Common Polymorphism in the Bile Acid Receptor Farnesoid X Receptor Is Associated with Decreased Hepatic Target Gene Expression Mol Endocrinol 2007 21 1769 1780 10.1210/me.2007-0025 17519356

[B56] Nijmeijer RM Gadaleta RM van Mil SW van Bodegraven AA Crusius JB Dijkstra G Hommes DW de Jong DJ Stokkers PC Verspaget HW Farnesoid X receptor (FXR) activation and FXR genetic variation in inflammatory bowel disease PLoS One 2011 6 e23745 10.1371/journal.pone.0023745 21887309 PMC3161760

[B57] Trauner M Halilbasic E Nuclear Receptors as New Perspective for the Management of Liver Diseases Gastroenterology 2011 140 1120 1125 e1112 10.1053/j.gastro.2011.02.044 21334334

[B58] Pittet V Juillerat P Mottet C Felley C Ballabeni P Burnand B Michetti P Vader JP Cohort profile: the Swiss Inflammatory Bowel Disease Cohort Study (SIBDCS) Int J Epidemiol 2009 38 922 931 10.1093/ije/dyn180 18782896

[B59] Becker T Knapp M A powerful strategy to account for multiple testing in the context of haplotype analysis Am J Hum Genet 2004 75 561 570 10.1086/424390 15290652 PMC1182044

[B60] Becker T Schumacher J Cichon S Baur MP Knapp M **Haplotype interaction analysis of unlinked regions** Genet Epidemiol 2005 29 313 322 10.1002/gepi.20096 16240441

